# Construction and analysis of a *Noccaea caerulescens* TILLING population

**DOI:** 10.1186/s12870-022-03739-x

**Published:** 2022-07-22

**Authors:** Yanli Wang, David E. Salt, Maarten Koornneef, Mark G. M. Aarts

**Affiliations:** 1 Laboratory of Genetics, Wageningen University & Research, Droevendaalsesteeg 1, 6708 PB Wageningen, The Netherlands; 2 College of Horticulture Science & Technology, Hebei Normal University of Science & Technology, No 360, West of HeBei street, Qinhuang Dao, China; 3 Future Food Beacon of Excellence & School of Biosciences, University of Nottingham, Sutton Bonington, LE12 5RD UK

**Keywords:** TILLING, Forward and reverse genetics, Ionomics, Metal toxicity, Zinc deficiency, Brassicaceae

## Abstract

**Background:**

Metals such as Zn or Cd are toxic to plant and humans when they are exposed in high quantities through contaminated soil or food. *Noccaea caerulescens*, an extraordinary Zn/Cd/Ni hyperaccumulating species, is used as a model plant for metal hyperaccumulation and phytoremediation studies. Current reverse genetic techniques to generate mutants based on transgenesis is cumbersome due to the low transformation efficiency of this species. We aimed to establish a mutant library for functional genomics by a non-transgenic approach, to identify mutants with an altered mineral profiling, and to screen for mutations in *bZIP19*, a regulator of Zn homeostasis in *N. caerulescens*.

**Results:**

To generate the *N. caerulescens* mutant library, 3000 and 5000 seeds from two sister plants of a single-seed recurrent inbred descendant of the southern French accession Saint-Félix-de-Pallières (SF) were mutagenized respectively by 0.3 or 0.4% ethyl methane sulfonate (EMS). Two subpopulations of 5000 and 7000 M2 plants were obtained after 0.3 or 0.4% EMS treatment. The 0.4% EMS treatment population had a higher mutant frequency and was used for TILLING. A High Resolution Melting curve analysis (HRM) mutation screening platform was optimized and successfully applied to detect mutations for *NcbZIP19,* encoding a transcription factor controlling Zn homeostasis. Of four identified point mutations in *NcbZIP19*, two caused non-synonymous substitutions, however, these two mutations did not alter the ionome profile compared to the wild type. Forward screening of the 0.4% EMS treatment population by mineral concentration analysis (ionomics) in leaf material of each M2 plant revealed putative mutants affected in the concentration of one or more of the 20 trace elements tested. Several of the low-Zn mutants identified in the ionomic screen did not give progeny, illustrating the importance of Zn for the species. The mutant frequency of the population was evaluated based on an average of 2.3 knockout mutants per tested monogenic locus.

**Conclusions:**

The 0.4% EMS treatment population is effectively mutagenized suitable for forward mutant screens and TILLING. Difficulties in seed production in low Zn mutants, obtained by both forward and reverse genetic approach, hampered further analysis of the nature of the low Zn phenotypes.

**Supplementary Information:**

The online version contains supplementary material available at 10.1186/s12870-022-03739-x.

## Background

Recently, genome sequencing of many plant species [1] has led to the availability of a large number of gene sequences in public databases which subsequently has encouraged the development of Reverse Genetics approaches. For this, one tries to associate the mutation of a gene with a non-wild-type phenotype in order to understand the function of the gene in the wild type. Especially in the model species *Arabidopsis thaliana*, random insertional mutagenesis approaches combined with sequencing of the insertion sites [2, 3], RNA interference-mediated gene silencing (RNAi )[4], genome engineering by CRISPER-Cas syste m[5–7], the derived precise gene editing-single base pair replacemen t[8, 9], and screening of mutations after chemical [10, 11] or physical irradiation mutagenesis [12] by Targeting Induced Local Lesions In Genomes (TILLING) have been used as reverse genetic platforms. However, except for TILLING, these techniques are all based on efficient *Agrobacterium tumefaciens*-mediated transformation, which limits their use because transformation efficiencies are very much species, sometimes even genotype, specific [13, 14].

*Noccaea caerulescens (J. & C. Presl*) F.K. Meyer (formerly known as *Thlaspi caerulescens*) is a diploid species from the Brassicaceae family, exhibiting extreme tolerance to zinc (Zn), nickel (Ni), cadmium (Cd) and/or lead (Pb) exposure as well as the ability to hyperaccumulate these metals in its leaves [15–19]. Together with the Zn/Cd hyperaccumulator species *Arabidopsis halleri*, *N. caerulescens* is one of the most prominent plant model systems to study the physiological and molecular basis of metal hyperaccumulation and tolerance [18, 20, 21]. *N. caerulescens* has received special attention because of its potential application as a source of genes that can be used to engineer phytoremediation or improve food micronutrient content through biofortification [22–24]. Although *N. caerulescens* is a frequently studied metal hyper-accumulator model species, the use of *N. caerulescens* has been hampered due to limited reverse genetic resources.

The transformation efficiency is very low in *N. caerulescens* [23, 25], which makes T-DNA based insertional mutagenesis cumbersome. Alternatively, the functional study of candidate genes in *N. caerulescens* could be achieved by using *Agrobacterium rhizogenes*-mediated root transformation, which has a high efficiency (about 50–90%) but provides non-heritable, chimeric mutants and is thus limited to the analysis of gene functions in the root but not any other parts of the plant [26, 27].

A mutant population has previous been generated for *N. caerulescens* by fast neutron mutagenesis [28]. In that experiment, the genetically diverse seeds of *N. caerulescens* collected in the field, in the vicinity of Ganges in the south of France, have been mutagenized aiming at identifying genetically stable, faster cycling lines for future genetic studies. The use of fast neutrons will often result in deletions, ranging from a single base pair (bp) to hundreds of kilo base pairs (kbp). Mutants with small deletions are very useful for gene functional study, but large deletions are likely to not only knock out the target gene but also several of the neighbouring genes, which will make it more difficult to match a mutant phenotype to the relevant gene [12]. Furthermore, many genes are essential, and deletions in these genes will pre-dominantly cause lethality, necessitating the generation of less severe mutations to understand gene function in *N. caerulescens*.

Chemical mutagenesis, such as with the classical mutagen Ethyl Methane Sulfonate (EMS), induces predominantly point mutations randomly distributed over the genome. With EMS a high degree of mutational saturation can be achieved without excessive DNA damage [29]. The main advantage of chemical mutagenesis is the ability to accumulate an allelic series of mutants with a range of modified functions, from wild-type to loss-of-function mutants. The latter may occur when EMS generates a premature stop codon, a frame shift mutation, mutations in essential amino acids or mutations that disturb intron splicing [30]**.** Such allelic series are desirable as they generate a wide repertoire of phenotypes, which provide more insight into the function of a single target gene. Additionally, chemical mutagenesis does not rely on genetic transformation or tissue culture regeneration techniques, thus it can be applied to any plant species, regardless of ploidy level, genome size, or genetic background [31]. An individual plant that carries a point mutation in a gene of interest can be detected in a mutagenized population through TILLING [32]. Since the inception of TILLING in *A. thaliana* [32], this method has been very widely applied to agronomic crops and animals [29, 31, 33, 34]. It uses post-PCR techniques to identify single bp mutations such as Li-Cor and the recently developed High Resolution Melting (HRM) curve analysis [31, 35, 36] and DNA re-sequencing [37]. In the traditional approach, PCR amplification is followed by the cleavage of mismatches using endonucleases such as CEL1 or ENDO1, that recognize single DNA strands [38, 39], and detect the mutants on the sensitive Li-Cor gel analyser [40]. In the HRM platform, PCR is followed by the direct detection of mutations based on DNA melting curve analysis, thereafter confirmation by sequencing is used. This platform has been applied successfully to detect mutations in TILLING populations of tomato [41], wheat [42] and a clinically relevant human pathogen [43].

In this study, we describe the development of a TILLING population for *N. caerulescens* based on the Saint-Félix-de-Pallières (SF) accession. This accession, also from the south of France, was previously identified as one showing the most promise as model metal hyperaccumulator [23, 44]. The effectiveness of EMS on plant growth and seed production was evaluated in the M1 and M2 generations. Based on the element concentrations in leaves, determined for all M2 individuals, putative mutants with disturbed ionome profiles were identified. Based on the occurrence of obvious morphological mutant phenotypes, we evaluated the mutation frequency. Moreover, for TILLING, the HRM curve-based mutation detection platform has been optimized by known point mutations in *Flower Locus C (FLC)* and applied to detect mutant alleles for the *Basic Leucine Zipper 19* (*bZIP19*) gene, encoding an important transcription factor for Zn homeostasis in plants. In *Arabidopsis thaliana*, this gene acts together with the *bZIP23* gene in the control of Zn deficiency responsive gene expression of Zn transporters, and other genes involved in early response to Zn deficiency [45]. Recently, these two bZIP transcription factors were also found to act as Zn sensors in *A. thaliana*, to control plant Zn status [46]. Orthologues of these genes are found in *N. caerulescens*, with only the *NcbZIP19* gene found to be expressed [47]. The genetic and molecular characterization of two early flowering mutants (*flc, svp*) from this experiment is described by Wang et al., 2020 [48]. This mutant population is the first TILLING population for *N. caerulescens* and can be an attractive genomic tool for research in plant development as well as a valuable resource for identifying novel genes that control ionome composition in this metal hyperaccumulating species.

## Results

### EMS mutagenesis of *Noccaea caerulescens* accession SF

**Fig. 1:**
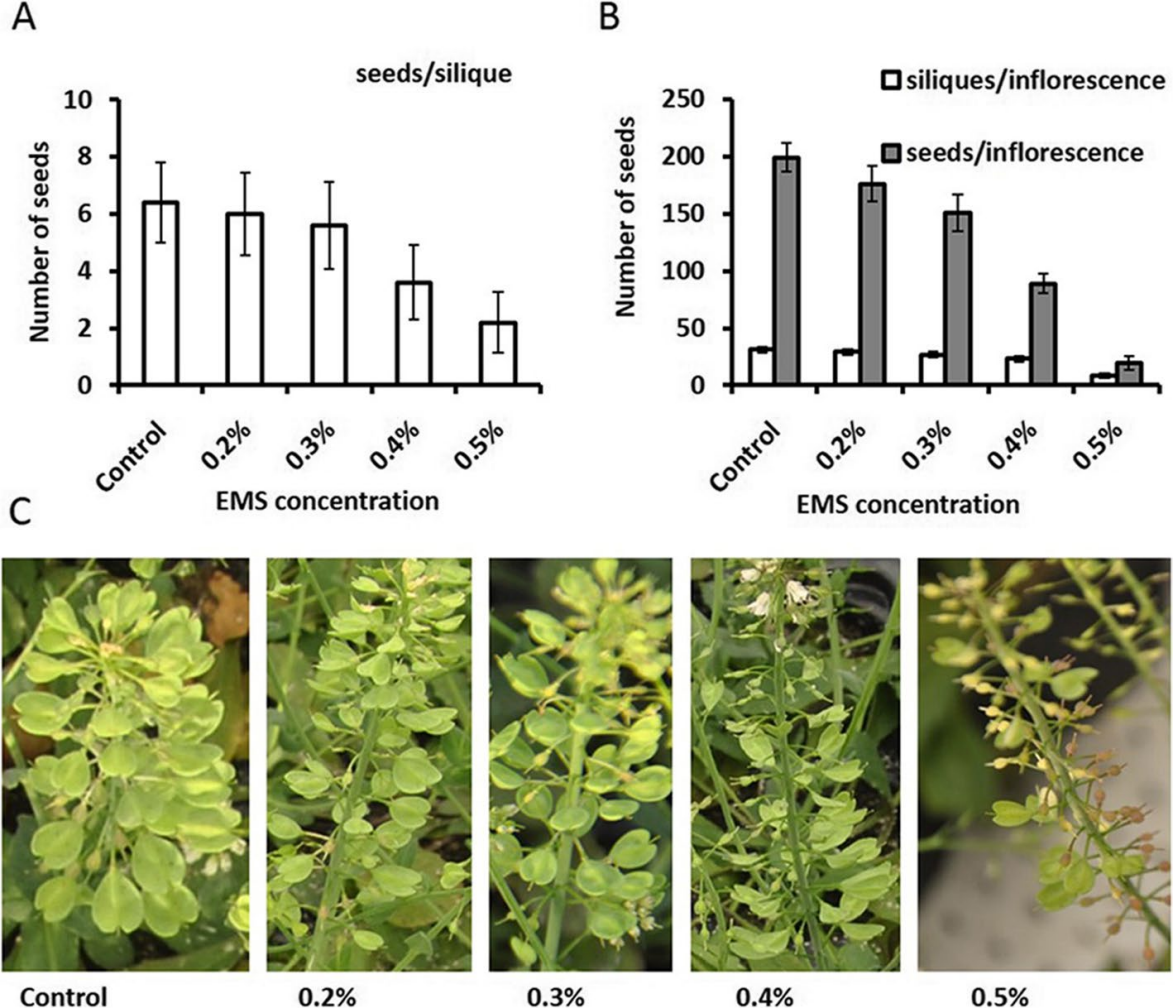
Optimization of the EMS concentration for *N. caerulescens* mutagenesis. Seeds were imbibed with four different ethyl methane sulfonate (EMS) concentrations and a control treatment with only distilled water (control) for 16 hrs in the dark. Plants were grown from these seeds, vernalized and allowed to flower upon which different traits were determined. **A** The number of seeds per silique, the number of siliques per inflorescence, and the number of seeds per inflorescence of seeds from different EMS treatments, presented in grey, white and dark bars. Data show the average of 3–5 plants ± SE. **B** Representative examples of the inflorescences of plants treated with the indicated EMS concentration.

To determine a suitable EMS concentration for mutagenesis of *N. caerulescens*, 200 seeds of an inbred line of *N. caerulescens* accession ‘Saint-Félix-de-Pallières’ (SF), a Zn/Cd hyperaccumulating accession, were soaked in different EMS concentrations (0.2, 0.3, 0.4 and 0.5%). A subset of the germinated seedlings from the 0.2 and 0.5% EMS concentrations were grown to evaluate their effectiveness for mutagenesis. The number of siliques and the number of seeds per silique in the M1 plants were determined (Fig. 1). At the higher concentrations of EMS, a stronger negative effect on fertility was observed, especially obvious at 0.5% EMS. At that concentration also the number of seeds per silique was negatively affected with only approximately 2–3 seeds per silique being produced. A reduction in seed number was already observed at 0.4% EMS, indicating 0.4% EMS was an effective concentration for mutagenesis in the SF accession of *N. caerulescens*. The use of 0.3% EMS was also expected to provide some effect, and resulted in a better fertility. Therefore the 0.3 and 0.4% EMS treatments were used to generate an M1 mutant population of *N. caerulescens*.

### Development of an M2 population in SF background

**Fig. 2:**
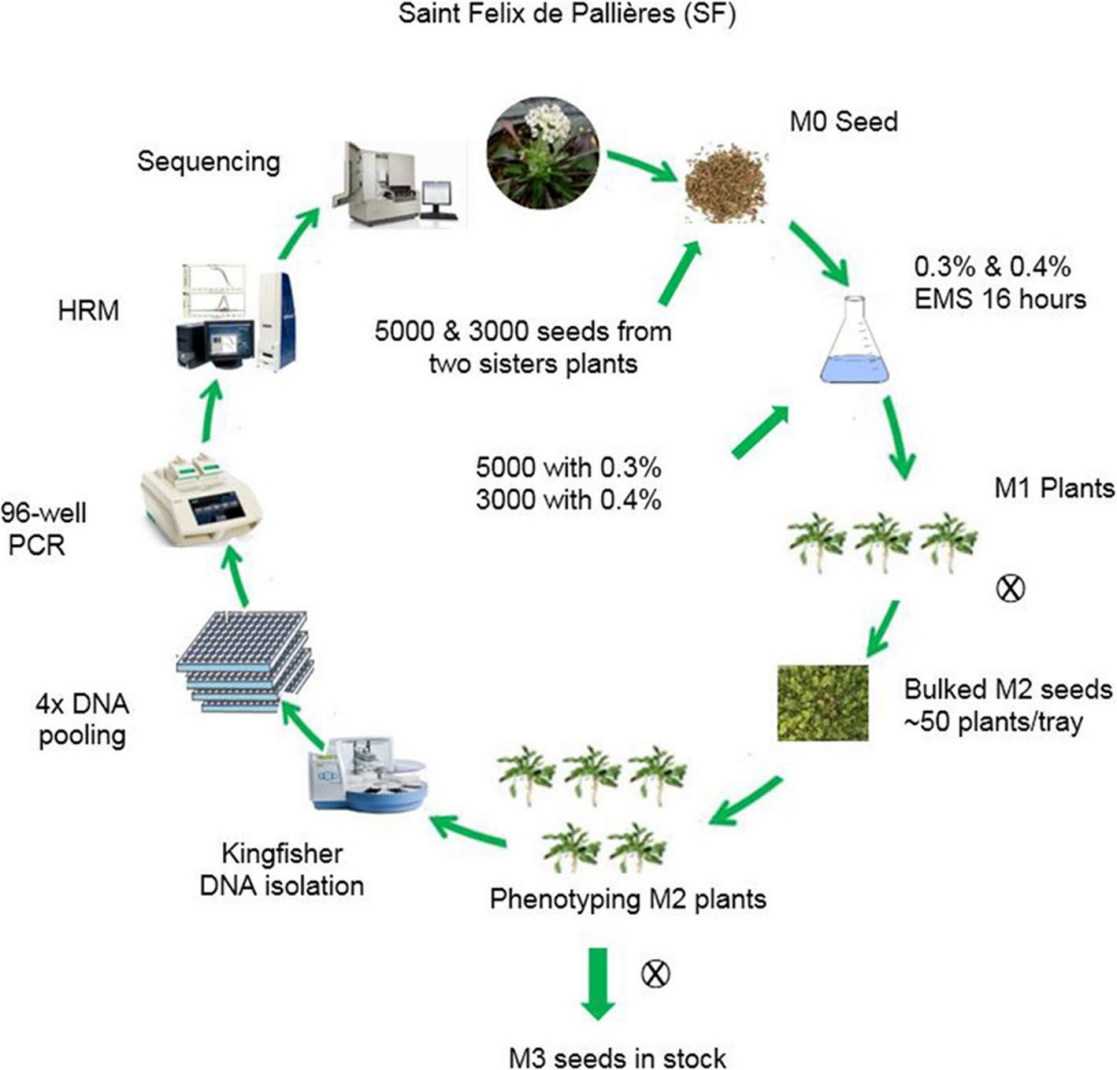
A schematic overview of the mutagenesis and TILLING procedure. M0 seeds from two sister plants of a single-seed recurrent inbred descendant of *N. caerulescens* accession Saint-Félix-de-Pallières were treated with 0.3 or 0.4% EMS to generate M1 seeds. These were sown in trays with 54 plants each. M1 plants were grown and allowed to self-fertilize to generate M2 seed, which was bulk-harvested per tray. A subset of the M2 seeds harvested per tray were sown to grow M2 plants in the greenhouse. In total 5520 and 7000 M2 plants were grown to obtain the 0.3 and 0.4% populations, respectively, the latter of which was used for TILLING. The M2 generation was phenotyped for plant morphological traits and their ionome profile. M3 seeds were harvested from individual M2 plants of the TILLING population. For TILLING by to High Resolution Melting curve analysis (HRM), the genomic DNA of individual plants was isolated using a Kingfisher DNA isolation robot. DNA of four plants was pooled in single wells of 96-well microtiter plates. Each pool was used for PCR amplification. PCR products per pool were subjected to HRM. The wells that contained mutations were selected and subjected to a second HRM screen. Positive samples were again PCR amplified and confirmed by DNA sequencing the PCR product.

To create an M2 population that can be used for TILLING, seeds of two sister plants of the SF accession were treated with 0.3 or 0.4% EMS to create two batches of M1 plants, grown in 148 trays of 54 pots per tray, 5000 plants for the 0.3% EMS treatment and 3000 plants for the 0.4% EMS treatment. Not all plants germinated or flowered, leaving around 50 plants per tray, from which M2 seeds were bulk-harvested for each of the 148 M1 trays. 120 M2 seeds per M1 tray were sown in individual pots to generate a population of ~ 7000 flowering M2 plants for the 0.4% EMS treatment, which was used for TILLING, and ~ 5500 flowering M2 plants for the 0.3% EMS treatment, which was used as back-up. Figure 2 represents the workflow and procedure followed to generate and sample the TILLING population.

### Morphological phenotypes in the mutagenized population

**Fig. 3:**
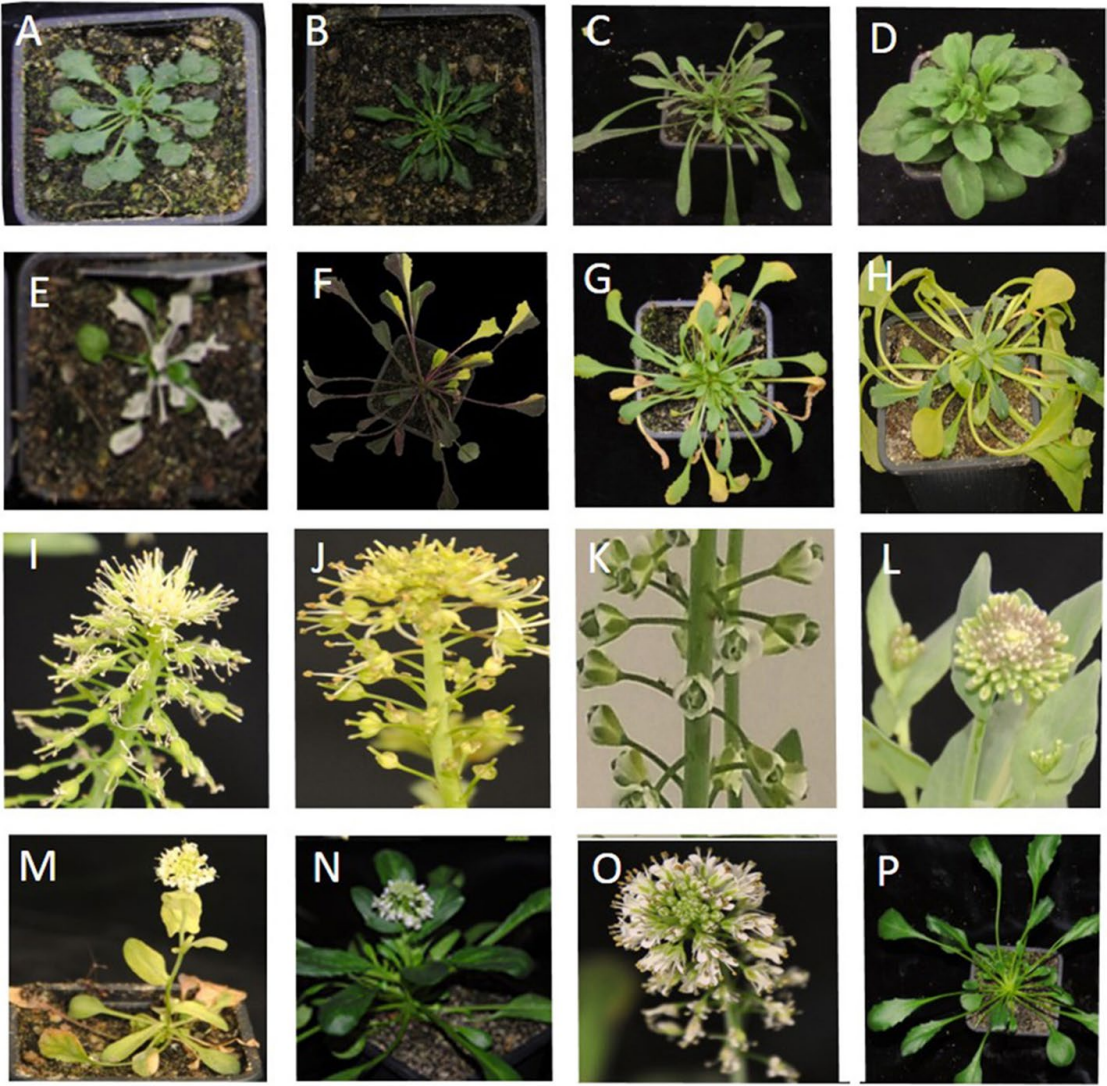
Examples of mutant phenotypes in the M2 population. Examples of morphological mutants illustrating the mutation spectrum observed in the *N. caerulescens* M2 populations. **A-D** abnormal leaf shape and organization; **E-H** chlorophyll phenotypes; **I-J** *apetala*-like phenotype; **K** *agamous*-like” double flower” phenotype; **L** *cauliflower*-like phenotype; **M** no side shoots, **N** non-vernalisation requiring early flowering mutant, **O** wild-type SF inflorescence, **P** wild-type SF rosette (2 months old). Additional mutant phenotypes are described in Supplementary Fig. S1.

To evaluate the effectiveness of mutagenesis, a broad range of visual morphological phenotypes were monitored in both populations, during the vegetative and reproductive stages. Mutants for plant architectural traits, such as leaf shape, leaf colour, inflorescence number, flower development and flowering time were observed in the M2 population. The most commonly observed phenotypes in both populations were related to anthocyanin pigmentation. The second largest class of mutants is related to leaf chlorophyll pigmentation ranging from narrow yellow spots, necrotic spots and yellow sectors (variegation), or generally lighter or darker green plants. On average, the frequency of chlorophyll pigmentation mutations was 1.2 and 2.1% in the 0.3 and 0.4% EMS treatment populations, respectively, further referred to as the 0.3 and the 0.4 populations. Flower morphology mutants were investigated upon flowering of the plants. 1.9% of the plants in the 0.4 population showed altered flower morphology phenotypes. *Eceriferum* mutants, with a bright green appearance due to an aberrant cuticular wax layer, and plants without secondary shoots account for 0.11 and 0.04%, respectively, in the 0.4 population. Mutants with altered wax layer, flowers with smaller petals and stamens and with multiple petals (double flowering) were exclusively observed in the 0.4 population, accounting for 0.18% in total. The most striking phenotypes are listed in Table 1. As expected, the 0.4 population yielded a higher percentage of mutant phenotypes than the 0.3 population. A selection of morphological phenotypes observed in the M2 populations are presented in Fig. 3. Many more other abnormal phenotypes were observed at different development stages, as listed in Supplementary Fig. S1. The frequency and the wide range of the phenotypic mutations observed in these two populations implied that especially the 0.4 population would be a valuable resource for screening desired *N. caerulescens* mutations in forward and reverse genetic screens.

**Table 1:** Numbers (#) and frequencies of indicated mutant phenotypes frequently observed in the 0.3 and 0.4% EMS treatment M2 populations. The frequency was calculated with respect to the total number of plants in each population

	phenotypes	# mutants		frequency (%)	
		0.3%	0.4%	0.3%	0.4%
leaf colour	anthocyanin	105	158	1.91	2.26
	chlorophyll	76	144	1.20	2.06
flower morphology	late flowering	15	50	0.27	0.71
	fasciation	17	40	0.31	0.57
	‘loose’ flower	3	10	0.05	0.14
	*terminal flower-like*	2	8	0.04	0.11
	*apetala-like*	2	8	0.04	0.11
	*cauliflower-like*	1	6	0.02	0.09
	early flowering	2	5	0.04	0.07
	no petals and stamens	0	3	0	0.04
	fused petals	1	2	0.02	0.03
	*agamous-like*	0	2	0	0.03
stem colour	*eceriferum-like*	0	8	0	0.11
architecture	no side shoots	1	3	0.02	0.04

### Optimization of the high resolution melting curve analysis

DNA samples were prepared from 7000 fertile M2 individuals of the 0.4 population. Samples from four M2 individuals were combined (4x pooling). To reduce cost and work load for screening the mutants in an efficient way, the High Resolution Melting Curve (HRM) analysis was optimized based on the analysis of a known EMS-induced point mutation in the *Flowering Locus C (FLC)* gene of *N. caerulescens* [45]. For this test, DNA samples of the *flc-1* mutant*,* carrying a G to A mutation (483 bp after the ATG start codon) were spiked into five wells in a 96-well plate, which contained the 4x pooled DNA samples from wild-type plants. The initial *FLC* HRM assay of this plate yielded seven positive samples that displayed aberrant melting curves indicating PCR fragments carrying points mutations in these wells (Fig. S2). After the second round of screening, four of the seven samples were confirmed. All four samples contained the spiked *flc-1* mutant DNA, meaning that only one spiked sample got undetected, but no false positives were found.

### Mutation of the *NcbZIP19* gene

**Fig. 4:**
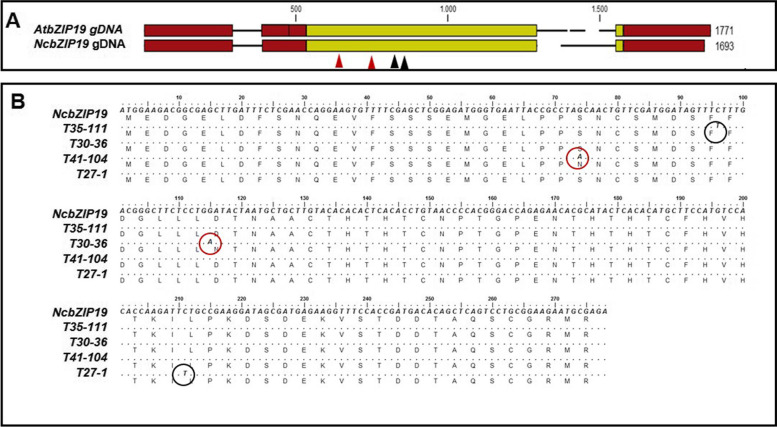
Sequencing results of four mutants for NcbZIP19. **A** Genome organization of the bZIP19 genes of *A. thaliana* (At) and *N. caerulescens* (Nc). Untranslated regions are indicated with red boxes, exons are indicated with yellow boxes, introns are indicated with black horizontal lines. The EMS-induced mutations are indicated by red (non-synonymous) and black (synonymous) arrows below the first exon. **B** Point mutations confirmed by sequencing. The two non-synonymous substations are shown in the red circles. Two synonymous substitutions are shown in black circles.

In order to study if a mutation of *NcbZIP19* affects the function of the gene and the expression of Zn uptake transporters, we screened the M2 population for mutants in *NcbZIP19*. In the whole TILLING population, 28 samples were identified as positives in the first round of HRM screening. After the second round of screening, 14 of these were selected as putative mutants. Four mutant alleles in *NcbZIP19* were identified after subsequent confirmation by sequencing HRM-positive PCR amplicons (Fig. 4B). Of these four mutations, all in exons, two encoded for non-synonymous amino acid substitutions, leading to a change in the predicted amino acid sequence, while the other two caused synonymous changes, which are unlikely to change the function of *NcbZIP19*. Of the two nonsynonymous mutations, plant T41–104 carried a G to A mutation at position 78 of the coding region, leading to a serine (Ser) to asparagine (Asn) amino acid substitution. In plant T30–36, a G to A mutation at position 115 of the coding region caused a substitution from aspartic acid (Asp) to asparagine (Asn). The two synonymous mutations were located at positions 196 and 211, respectively in the coding region. Figure 4A shows a schematic representation of the *NcbZIP19* gene, marking the location of the induced mutations.

### Genome-scale mutant frequency

The frequency of visible phenotypes in a mutant population indirectly reflects the overall, genome-wide mutation frequency. To determine the chance of obtaining a target mutation with an obvious phenotype in the TILLING population, we evaluated the mutation frequency based on the observation of visually mutant phenotypes that correspond to mutations in supposedly or confirmed single genes. A total of five early flowering and two double flower mutants were identified (Table 1). Of the early flowering mutants, we identified two mutations in the *FLC* gene and two in the *SVP* gene [36]. For the *flc-1* and *flc-2* mutants, which are identical, the G to A point mutation is located exactly at the exon-intron junction at the 3′ end of the third exon (345 bps after the ATG start codon), causing aberrant splicing leading to a truncated protein. The G to A point mutation in the *flc-3* mutant is located at the intron-exon junction at the 5′ end of the third exon (286 bps after the ATG start codon). This mutation also causes aberrant splicing, which results in a 9-bp in-frame deletion in the mRNA at the very beginning of the third exon. The two other early flowering mutants were due to mutations in the *SVP* gene, producing respectively an in-frame deletion in the fourth exon due to aberrant splicing (G to A mutation) and a non-synonymous leucine to phenylalanine substitution in the first exon (C to T mutation). The double flower phenotype is most likely due to mutations in the *AGAMOUS* (*AG*) gene [49], which is a single copy gene in *A. thaliana* and *N. caerulescens*, but this is not confirmed. Two to three mutants for each of the three monogenic phenotypes suggests that mutant saturation was almost complete in the TILLING population, with a chance of 13.5% to find no mutants for a gene of comparable size (~ 700 bps in ORF) in this population, based on a Poisson distribution. Based on the two to three mutants obtained for these three genes (*FLC*, *SVP* and *AGAMOUS)*, we could estimate the number of knockout mutations per plant in the TILLING population. Assuming one mutant/3000 M2 plants, and around 30,000 genes (29,712) to be predicted for *N. caerulescens* [47], we expect every M2 plant to carry on average knock-out mutations in 10 genes.

### Identification of ionome mutants

**Fig. 5:**
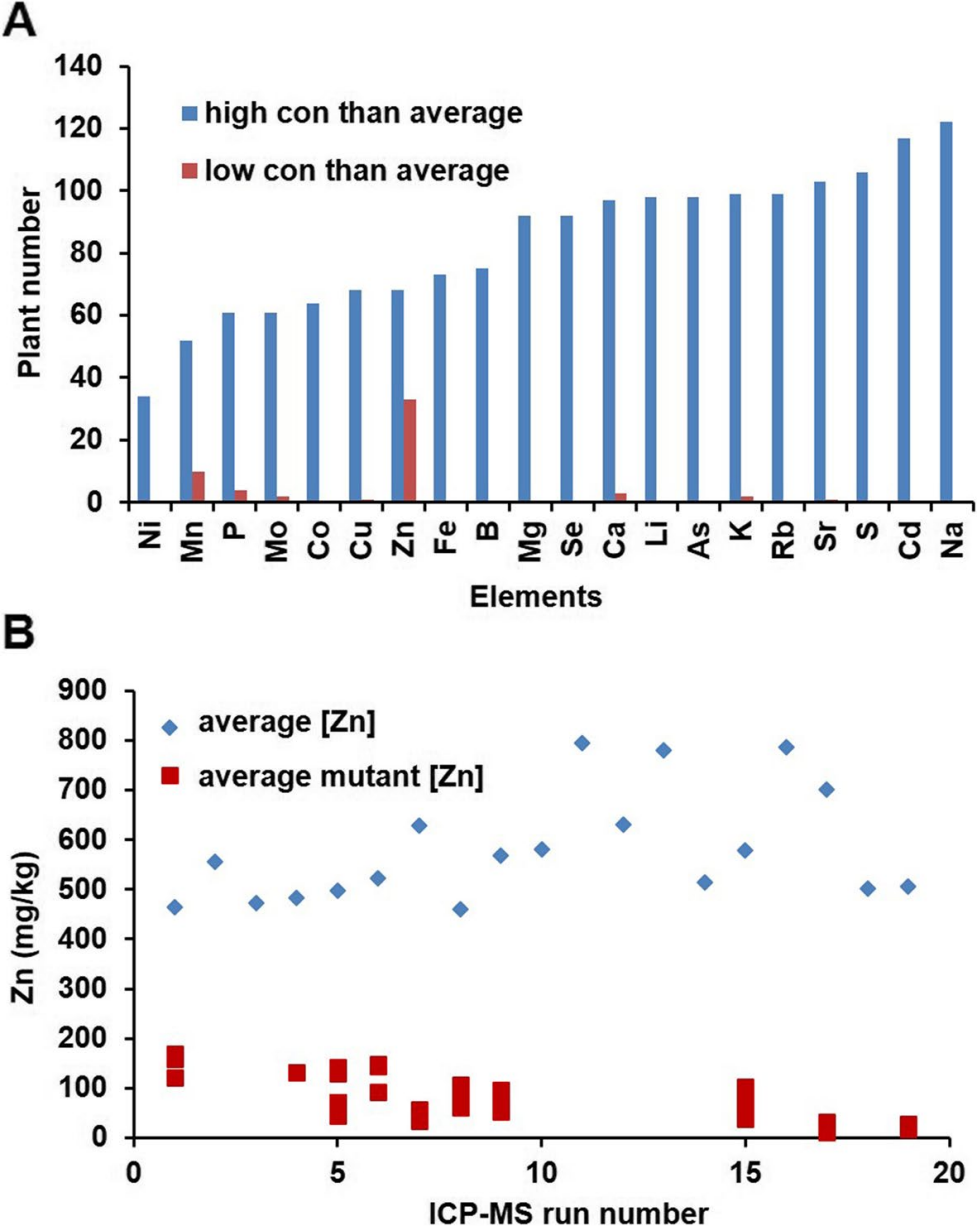
Number of putative ionome mutants in the *N. caerulescens* M2 population. **A** Putative ionome mutant numbers identified in the 0.4% EMS treatment M2 population, consisting of ~ 7000 plants, based on Z-score values (Z ≥ +/− 3) to have either a higher (blue) of lower (red) concentration (con) than average for at least the indicated element. Mutant numbers are indicated per ICP-MS run, ordered from lowest to highest number of mutant plants. **B** Distribution of putative low-Zn mutants in the M2 population. The 7000 M2 plant ionomes were determined in 20 different runs by ICP-MS. Blue diamonds indicate the average Zn concentration for each run. Red squares indicate the Zn concentration of putative low-Zn mutants in each run.

Considering that *N. caerulescens* is a Zn/Cd/Ni hyperaccumulator, leaves of all M2 plants were sampled to determine their leaf ionome by Inductively Coupled Plasma Mass Spectrometry (ICP-MS). Plants in which one of the 20 trace elements that were analysed (Li, B, Na, Mg, P, S, K, Ca, Mn, Fe, Co, Ni, Cu, Zn, As, Se, Rb, Sr, Mo and Cd) was found to have a concentration that was three or more standard deviations higher or lower than the concentration in the WT (Z-score analysis) were considered to be putatively high or low element mutants [50] (Fig. 5A). Since no Ni or Cd was supplied to the plants, we initially focused on the identification of low Zn mutants in the 0.4 population. In total, 34 putative low Zn mutants were identified in this M2 population. These mutants were found in different seed batches, suggesting they arose from independent mutation events (Fig. 5B).

**Fig. 6:**
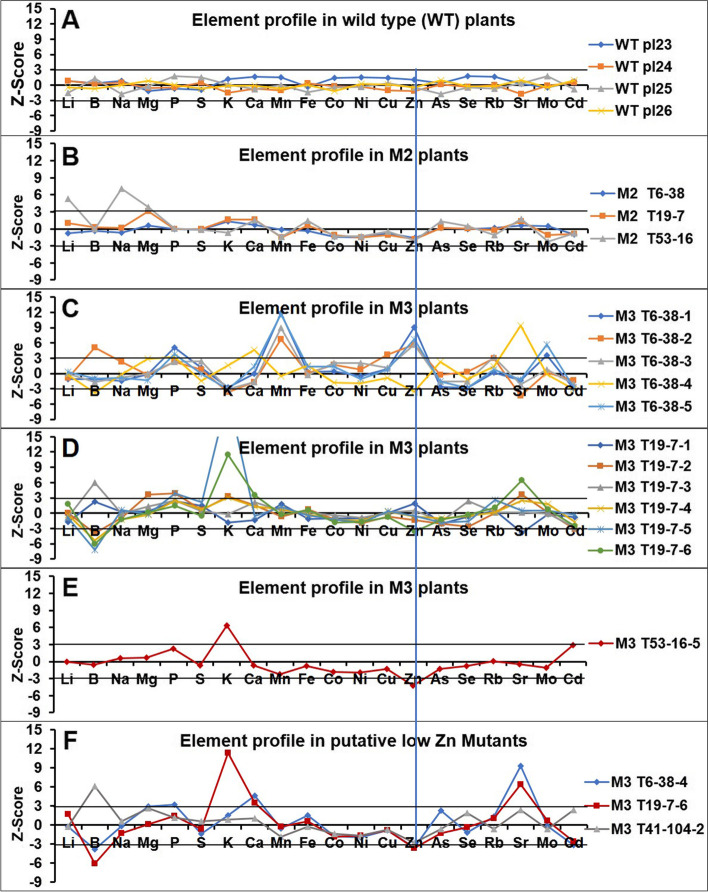
Leaf ionome profiles of representative putative *N. caerulescens* low-Zn mutants. Ionome profiles based on ICP-MS analysis of one mature expanded leaf per M2 or M3 plant, indicating the Z-scores for each element of indicated plants. The Z-scores for M2 plants were normalized to the average of 7000 individuals. The Z-score for M3 plants were normalized to the average of 50 M3 plants tested. The ionome profiles are shown for (**A**) SF wild-type (WT) plants; **B** low-Zn M2 plants; **C** M3 progeny of M2 T6–38; **D** M3 progeny of M2 T19–7; **E** M3 progeny of M2 T53–16; **F** putative low-Zn mutants selected from each M3 progeny.

To further confirm the element profile of the putative mutants, representative putative low Zn mutants T6–38, T19–7 and T53–16 were selected and 10–15 M3 progeny of those mutants were grown in the greenhouse. The ionome profiles of these progeny plants were not identical, which would be expected for progenies of recessive mutants (Fig. 6) and therefore may suggest (partial) dominance. Among the progeny of T6–38, plant T6–38-4 accumulated very little Zn but showed high Sr and Ca concentrations. The four sister plants, however, contained much higher Zn and Mn concentrations than the wild type plants. Plant T6–48-4 was much reduced in size compared to the other plants and displayed strong leaf chlorosis. None of the other putative mutants, neither in the same line nor in others, displayed any obvious aberrant morphological phenotypes (**data not shown**). As for the progeny of T19–7, plant T19–7-6 showed a very low Zn concentration, but a higher concentration of K and Sr. Most of the progeny of T54–16 did not grow, with only one plant to be alive when sampling. It confirmed the low Zn concentration of the M2 parent and showed a high K concentration. The element profiles of the progenies compared to their mother plant (M2) indicated that the element profiles of the M3 plants T6–38-4, T19–7-6 and T53–16-5 are similar to those found in the M2 generation.

**Fig. 7:**
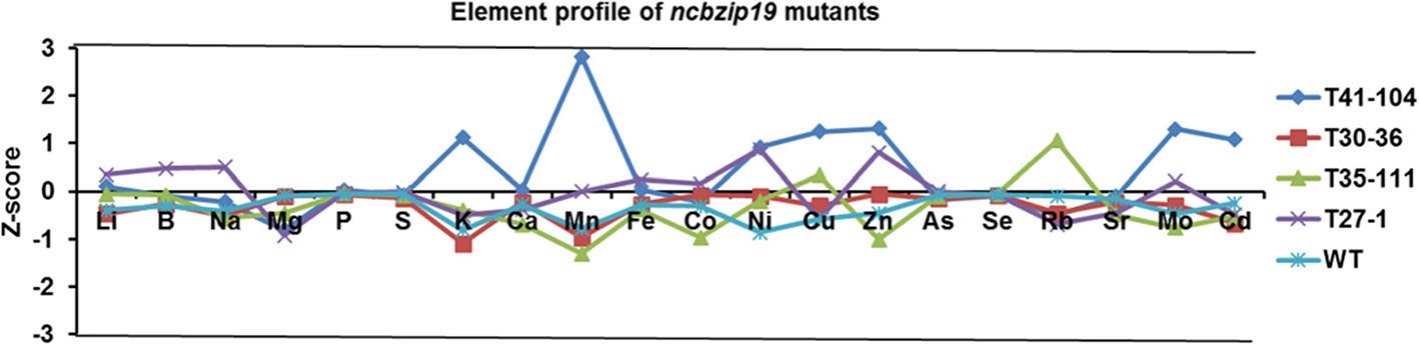
The ionome profiles of NcbZIP19 mutants and WT plants. Plants T41–104 and T30–36 contain each a mutation leading to a non-synonymous amino acid substitution in NcbZIP19. Plants T35–111 and T27–1 contain each a mutation leading to a synonymous substitution. Profiles of single M2 individuals for each NcbZIP19 mutant are shown. Profile data of WT is the average of six plants. Z-score: standard deviations difference from the means of WT.

The availability of the element profiles for the whole M2 population allowed us to check the ionome profiles of the two non-synonymous *ncbzip19* mutants, T41–104 and T30–36. According to the Z-score analysis, the element profiles of these mutants were not significantly different from the wild type (Fig. 7), although the concentrations of K, Ni, Cu, Zn, Mo, Cd and particularly Mn, were higher in T41–104 than in WT. Unfortunately, neither of these two mutants produced viable offspring (no seeds or no germinating seeds), which made further analysis of them impossible.

## Discussion

*Noccaea caerulescens* is a Zn/Cd/Ni hyperaccumulator, which has been used as a prominent model to study the molecular mechanism underlying metal hyperaccumulation [22]. However, the further use of *N. caerulescens* in research has been hampered by its recalcitrance to transformation, because of which reverse genetic resources are not available. The present study describes the generation of a mutant population of *N. caerulescens* by EMS-mutagenesis, a non-transgenic approach. The range and variety of morphologically aberrant and leaf ionome phenotypes observed in the M2 generation demonstrated that this population has been effectively mutagenized, which makes it a valuable resource to isolate mutants disturbed in elemental composition as well as plant morphology or any other relevant trait, including metal tolerance and hyperaccumulation. For reverse genetic purposes, the HRM analysis platform was optimized and successfully applied to screen this TILLING population for an allelic mutant series of the *bZIP19* transcription factor gene. The obtained results demonstrated that the population serves as an adequate resource for both forward and reverse genetics purposes.

The mutant frequencies in the *N. caerulescens* mutant population were evaluated based on the frequency of single gene morphological mutants identified in the M2 population. We expect that for every gene on average 2.3 knockout mutants can be found upon screening the TILLING population of 7000 M2 plants. A similar mutation frequency has been found in the early research in *A. thaliana* to provide suitable mutagenized populations [51]. An alternative procedure is to estimate the mutant frequency at the DNA level using targeted approaches also used for TILLING, such as Li-COR and HRM technology, or genome wide screening by Next Generation Sequencing (NGS) as reviewed by Schneeberger (2014). The genome-wide mutant frequency was evaluated by whole genome screening of mutations in a few representative targeted genes in e.g. *Arabidopsis thaliana* [52], *Brachypodium distachyon* [53], in rice (*Oryza sativa*), wheat (*Triticum aestivum L*) and tomato (*Solanum lycopersicum* L.) [30, 54–59]. The mutant frequencies for those species are 1/300 kb in Arabidopsis [60], 1/396 kb in *B. distachyon,* 1/451 kb in rice, 1/500 kb in *B. rapa* [57] and 1/737 kb in tomato [61]. Based on the mutant frequency, the number of the point mutants one plant carries was estimated, varying from a few to several hundred per plant e.g. around 1000/plant for *Medicago truncatula* [62], 10.000/plant in *B. rapa* [57] and 430/ plant in rice [63]. The evaluation of mutant frequency in these studies also include base pair mutations that did not cause obvious phenotypes. We could make a rough calculation of the knockout mutant frequency at genome level based on the loss-of-function mutations in the *FLC*, *SVP* and *AG* genes. The genome frequency of identifying the knockout mutations of a monogenic phenotype from our TILLING population was approximately one knockout mutant per 2000 kbp (The gene length screened x the total number of Tilling individuals / The total number of morphological mutants). This suggests that each M2 plant of this population will contain, on average, one bp mutation per 2000 kbp. This frequency, either calculated on sequence level or on phenotype level, is too low to guarantee a mutation in every gene, as there still is a 13% chance for ~ 700-bp target genes to not be mutated. Some genes may be smaller than 700 bp, and will have even less chance to be hit with a KO-inducing bp mutation. However, a higher mutation frequency could lead to more fertility problems than observed already, and a larger population will be logistically challenging for this species. The mutation density in a TILLING population depends upon various factors such as species, genotype, mutagen dosages and polyploidy [64]. Of those factors, mutagen concentration would be a very crucial one. Higher mutant density would be achieved with the higher EMS concentration applied, but the fertility will be also negatively affected. We concluded that 0.4% EMS is a suitable concentration to use for mutagenesis of the *N. caerulescens* SF accession, with the possibly to identify two to three loss-of-function mutations for a monogenic trait.

The HRM platform was used to identify potential mutation events based on a visual detection of aberrant HRM curves, meaning HRM curves that differ from the bulk of HRM curves. In order to detect mutations in *N. caerulescens* in an efficient and effective way, we adapted a 4x pooling strategy. This seemed adequate, as when we tested a known point mutation in the *FLC* gene we did not find false positives and only failed to detect the mutation in one pool (one false negative). To minimize false negatives, a large number of false positives were accepted in the first screen for the *NcbZIP19* gene. After the final appraisal of the mutations by sequencing of the amplicons, four *NcbZIP19* mutants were identified*. NcbZIP19* is expected to control the expression of *ZIP* and *NAS* genes in *N. caerulescens*, as has been found in *A. thaliana* [45]. Mutation of *NcbZIP19* is expected to reduce the expression of these target genes, and leading to a lower Zn hyperaccumulation. The two *Ncbzip19* mutants with non-synonymous substitutions did not display an altered ionome profile when compared to WT, suggesting that the consequence of these two nonsynonymous substitutions was neutral. In *A. thaliana*, *bZIP19* and *bZIP23* function redundantly to regulate Zn homeostasis. Under normal Zn supply, the Zn concentration is significantly lower in the root of *bzip19* and *bzip23* mutants compared to WT, but less prominent differences in Zn concentration are found in the shoot of *bzip23* [45, 65]. In *N. caerulescens*, the orthologous gene of *bZIP23* is hardly expressed and possibly not responsive or not involved in the response to zinc deficiency [47].

We also used the TILLING population for a forward genetic screen for ionome mutants. Identification of variation in element accumulation among putative *N. caerulescens* mutants provides an excellent starting point for identifying genes important for regulation of the plant ionome, and with regards to Zn and Cd, for regulation of Zn/Cd hyperaccumulation. We selected a few putative low Zn mutants as representatives, and partially confirmed their phenotype in the M3 generation. However, not all progeny showed the same low Zn phenotype as observed in the M2. This was unexpected, if assuming that the mutant phenotypes would be recessive, as is normally the case for EMS-induced point mutations [66]. One explanation for the observed phenotypic variation could be that outcrossing has occurred between the identified mutant and neighbouring plants. Although *N. caerulescens* is predominantly self-fertilizing with a cross-fertilization rate in the field ranging from 5 to 10% under normal growth conditions [67], this could be higher if either the growth conditions or the mutagenesis process caused some (male) sterility, as was described for sorghum [68]. Alternatively, the mutant itself could be partially self-sterile, not unlikely, since Zn is a key constituent of many enzymes and proteins, and Zn deficient plants are known to be partially sterile [69]. Especially if the species normally hyperaccumulates Zn, the inability to do so may have exacerbated any Zn deficiency phenotypes, not normally seen, even under Zn limiting conditions. In any case, we were not able to recover many, if any, seeds of these low Zn mutants, which supports the notion that partial sterility may be the consequence of the mutation. As an example, plant T38–4 was identified to have a low Zn accumulation, and it also appeared very small, poorly grown and with severe leaf chlorosis, hinting at a very severe Zn deficiency phenotype. The limited seed production complicated genotyping and phenotyping of putative low-Zn mutants in the M3 generation. To avoid losing potential mutants, more care should be taken when propagating the mutants for further functional analysis. The 0.3% EMS population showed a relatively higher fertility, but unfortunately with a lower mutant frequency, in comparison to the 0.4% EMS population, which makes it less attractive to include in the TILLING population.

Most of the *N. caerulescens* mutants profiled in this study had at least one element that was significantly different (Z-score) from the wild type, indicating that mineral homeostasis genes might be involved, or alternatively, that more than one gene controlling mineral compositions might be affected in those mutants. This would allow the identification of mutant series affecting virtually any gene of interest by TILLING. The identification of the novel genes for targeted traits is promising in this population, via forward techniques like map-based cloning [70, 71], or mapping by sequencing [72, 73]. This is the first functional genomics tool in *N. caerulescens*, an extraordinary Zn/Cd/Ni hyperaccumulating species. This mutant library will facilitate the application of functional genomics in *N. caerulescens* as well as comparative functional analysis with related species.

## Conclusions

The EMS mutant population reported here is the first mutant library established for *N. caerulescens,* which will be suitable for mutation identification by forward and reverse genetic approaches. 0.4% EMS is an effective concentration for *N. caerulescens* mutagenesis. The TILLING population size of ~ 7000 plants will on average yield 2–3 knockout mutations per candidate gene, assuming an ORF size of around 700 bp.

## Methods

### Plant materials and growth conditions

An inbred line of the calamine *N. caerulescens* accession ‘Saint-Félix-de-Pallières’ (SF) has been used for mutagenesis [45]. Seeds of the Saint-Félix-de-Pallières accession were obtained from Dr. Henk Schat [74], formerly Vrije Universiteit, Amsterdam, who collected some seeds of plants grown on a public road side leading towards the former Zn mining site in the vicinity of Saint-Félix-de-Pallières (44° 2′40.03“ N, 3°56’18.05” E) in the southern Cevennes, France, before 2010. A local resident overviewing the site provided permission and guidance in collecting the seeds. Dr. Schat also formally identified the plants grown from the seeds to be *Noccaea caerulescens*. To ensure the homogeneity of the seeds used for mutagenesis, the plants were self-fertilized for five generations by single seed descent (SSD). After EMS treatment (see below), all M1 plants were grown and allowed to spontaneously self-fertilise in an insect-free greenhouse between October 2012 to May 2013. Plants were grown in trays with 54 pots each, filled with a mixture of regular, fertilized, gardening peat and sand. Plants were watered twice a day. The temperature was above 18 °C during the night and around 23 °C during the day; complementary artificial lighting was provided to attain a 16-h day. Two months after sowing, the plants were naturally vernalized in a “cold” greenhouse (only heated to keep the minimum temperature above 4 °C), for two and half months (day temperature was between 5 and 10 °C, the night temperature varied between 4 and 6 °C). After vernalization, the plants were transferred to a heated greenhouse for flowering, under long day conditions (16-h day/8-h night) with (day/night) temperatures around 20–23 °C. In these greenhouse conditions, the duration of the growth cycle per generation is around 7 months. For the M2 generation, the plants were grown from October 2013 until June 2014 under the same culturing regime. All the plants used for this experiment were growing in the same greenhouse and under the same conditions.

### EMS mutagenesis

The mutagenesis scheme is outlined in Fig. 2. Seeds obtained from self-fertilization of two sister plants were treated with either a 0.3% or a 0.4% Ethyl Methane Sulfonate (EMS, Sigma-Aldrich) concentration, by following the EMS protocol for higher plant mutagenesis [75]. The seeds were imbibed in water for 16 hrs before being treated with 0.3% (~ 5000 seeds) and 0.4% (~ 3000 seeds) (v/v) of a freshly prepared EMS solution. After treatment, the seeds were washed at least 10 times with demi-water. After washing, the seeds were suspended in 0.15–0.2% [w/v] water agar solution and sown directly on soil in 148 trays (54 pots / tray) using a pipette. Six months after sowing (including two months of vernalisation), the M2 seeds from the ~ 50 remaining plants per tray were bulk harvested after removing the plants that had not produced seeds. For each M1 tray, 120 M2 seeds, bulk-harvested from all M1 plants in each tray, were sown in separate pots (7 × 7 × 8 cm) and were individually labelled. The plants were transferred to the “cold” greenhouse for vernalisation between end of December 2013 until mid of March 2014. Morphological and pigmentation characters were monitored at different development stages. After transfer to the heated greenhouse the non-flowering plants (~ 300) were removed from the M2 population, resulting in ~ 7000 individuals in the 0.4% EMS population for ionome analysis and TILLING. The M3 seeds from these plants were collected and are kept in long-term storage for future analysis. The 0.3% EMS population consisted of around 5000 individuals, which was used as back-up for TILLING.

### Element profiling by ICP-MS

To determine the shoot ionome profile, one mature leaf was harvested from each M2 individual when the 5-months-old plants were flowering. Samples were collected in 96-deep-well plates (QIAGEN) and dried for 72 h at 60 °C. The leaf material was acid-digested [50] with some modification: Because the amount of organic matter in the sample appeared to be more than for *A. thaliana*, a pre-digestion was carried out overnight in nitric acid/hydrogen peroxide, before we heated the samples for full digestion. Elemental analyses were performed using inductively coupled plasma mass spectrometry (ICP-MS; Elan DRC II; PerkinElmer, http://www.perkinelmer.com) to determine the concentrations of Li, B, Na, Mg, P, S, K, Ca, Mn, Fe, Co, Ni, Cu, Zn, As, Se, Rb, Sr, Mo and Cd as described by [50]. Putative mutants for any of the analysed elements were selected based on the Z-score value (three or more SD values higher or lower than WT )[50].

### DNA extraction and quantification

Genomic DNA was extracted from the top of the inflorescence of each plant in the 0.4% EMS M2 population. For each sample, around 50 mg of plant material was collected in 96-deep-well plates (QIAGEN) with two 4-mm glass beads in each tube and stored at − 80 °C until further use. Prior to the DNA isolation, the samples were deep-frozen in liquid nitrogen for one minute and homogenized twice using a Deep Well shaker (Vaskon 96 grinder, Belgium; http://www.vaskon.com) for 2 minutes at 16.8 Hz, thereafter following the Nucleo Mag Plant isolation protocol (http://www.mn-net.com/), the lysis buffer (300 μl) with RNase was added and samples were incubated in a water bath (65 °C) for 30 mins. After centrifugation for 10 mins at maximum speed, 300 μl supernatants were transferred to a new 96-deep-well plate, mixed with binding buffer (150 μl) and magnetic beads (30 μl). After this step, the prepared samples were ready for DNA isolation by KingFisher™ Flex Magnetic Particle Processors (Thermo) by following the protocol of the kit (http://www.mn-net.com/).

For the final step, the DNA was eluted by 100 μl Elution buffer, instead of 150 μl recommended by the protocol, to reduce contaminations. A few plates were randomly selected for evaluation of the DNA quality by Nanodrop and gel electrophoresis. The DNA samples were all highly homogenous and the DNA concentrations were comparable, at around 30–40 ng/μl, for the whole population. The OD260/280 ratios were around ~ 1.8 to 2.0, indicating good quality DNA. For mutation screening purposes, the DNA samples were normalized and 4 samples were pooled in 96-well format. 2 μl of pooled genomic DNA was used in multiplex PCRs for mutation detection analysis.

### PCR primer design and amplification

All PCRs were performed using FramStar 96-wells plates (4titude, UK, http://www.4ti.co.uk). Specific primer sets were designed for this study based on preliminary *N. caerulescens* whole genome sequences (of the Ganges accession) (manuscript in preparation) or on published cDNA sequences [47, 74] using Primer3plus (http://primer3plus.com/) software. The primer sequences are listed in Table S1. PCR fragments (~ 400 bp) obtained from a characterized EMS-induced early flowering *flc-1* mutant [36] were used to setup the HRM mutation detection platform. The PCRs were performed on 4× flat pools in FramStar 96-wells plates (4titude, UK, http://www.4ti.co.uk). HRM primer amplification efficiencies and specificities were determined by PCR amplification of wild-type SF DNA.

### High resolution melting (HRM) curve analysis

Positive pools were selected by analysing the melting temperature profiles. If the pool contains a mutation it showed a lower melting temperature than homogeneous DNA fragments. The positive pools were screened again by using a single DNA sample in each tube. Positive samples were confirmed by sequencing [35].

### Sequencing

Mutations were validated by DNA fragment sequence analysis. The PCR product was purified using the NucleoSpin® Gel and PCR Clean-up kit and purified fragments were sent for sequencing to Eurofin Genome Sequencing Company (Ebersberg, Germany) (https://www.eurofinsgenomics.eu/). The sequences and amino-acid sequences of *NcbZIP19* were aligned by using the Bioedit program (http://www.mbio.ncsu.edu/bioedit/bioedit.html). Identical amino acids are indicated by dots, the deletion of an amino acid was indicated with a dash.

## Supplementary Information

**Additional file 1.**

## Data Availability

The TILLING population and datasets generated and analysed during the current study are available from the corresponding author on reasonable request.

## Abbreviations

*bZIP*: Basic-Region Leucine-Zipper
HRM: High Resolution Melting
PCR: Polymerase Chain Reaction
ICP-MS: Inductively Coupled Plasma Mass Spectrometry
CEL1: Celery 1 enzyme
*FLC*: *Flowering Locus C*
TILLING: Targeting Induced Local Lesions In Genomes
EMS: Ethyl Methane Sulfonate
