# Supplementary material for: Construction and analysis of a Noccaea caerulescens TILLING population
Source: BMC Plant Biol. 2022 Jul 22;22:360. doi: 10.1186/s12870-022-03739-x (PMC9308233; doi:10.1186/s12870-022-03739-x)
Supplement: Supplementary file 1 — Additional file 1. [file 12870_2022_3739_MOESM1_ESM.docx]

## Supplementary files to Wang et al., Construction and analysis of a *Noccaea caerulescens* TILLING population

***Fig. S1***

***
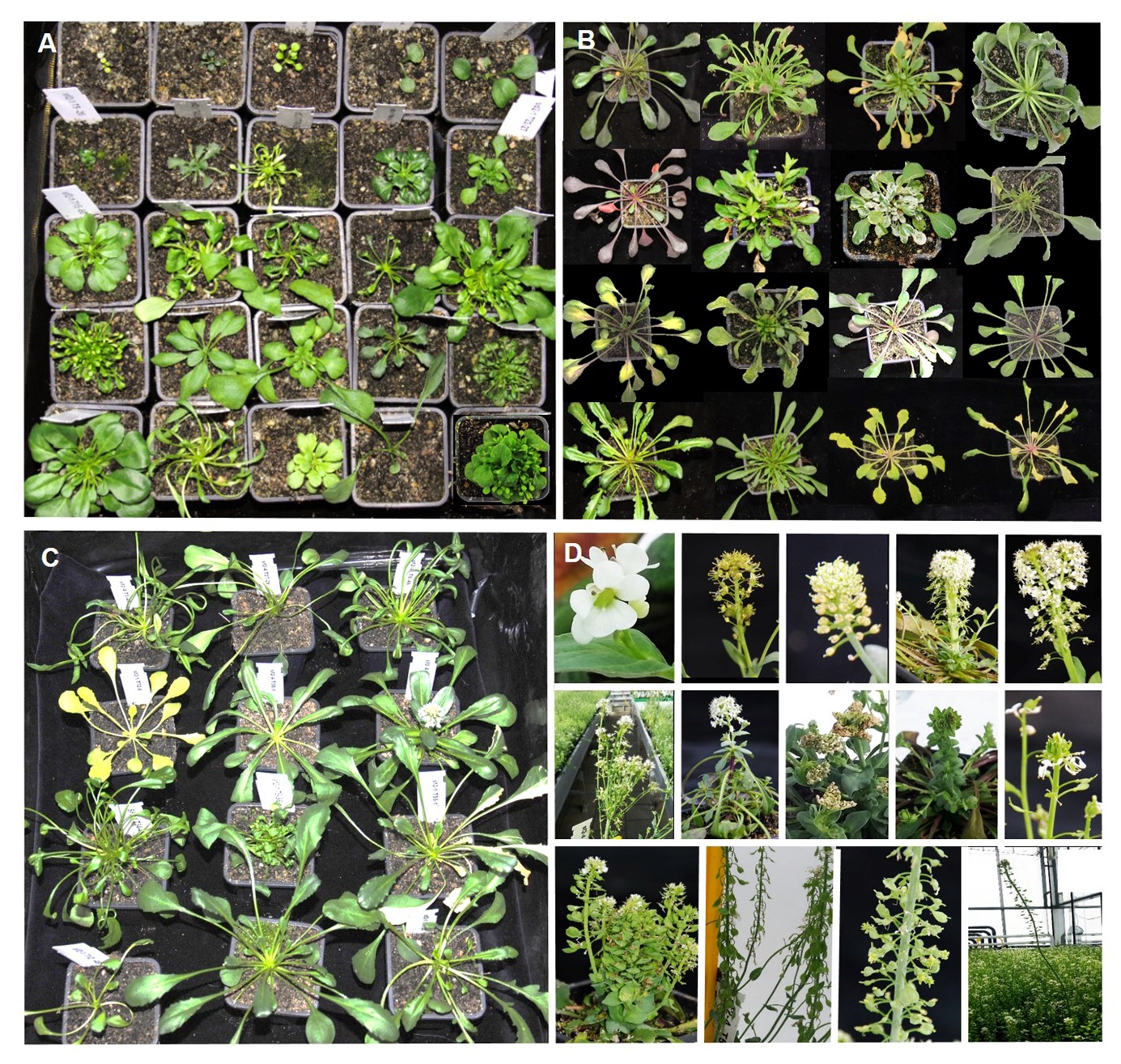
***

***Fig S1 Additional morphological mutations identified in the M2 generation in vegetative growth stage.*** *A: abnormal plant size and leaf shape; B: chlorophyII aberration; C: Plants with curly* *leaf petiole; D: abnormal organization of flowers, inflorescences, siliques and a super tall plant.*

***Fig. S2***

*
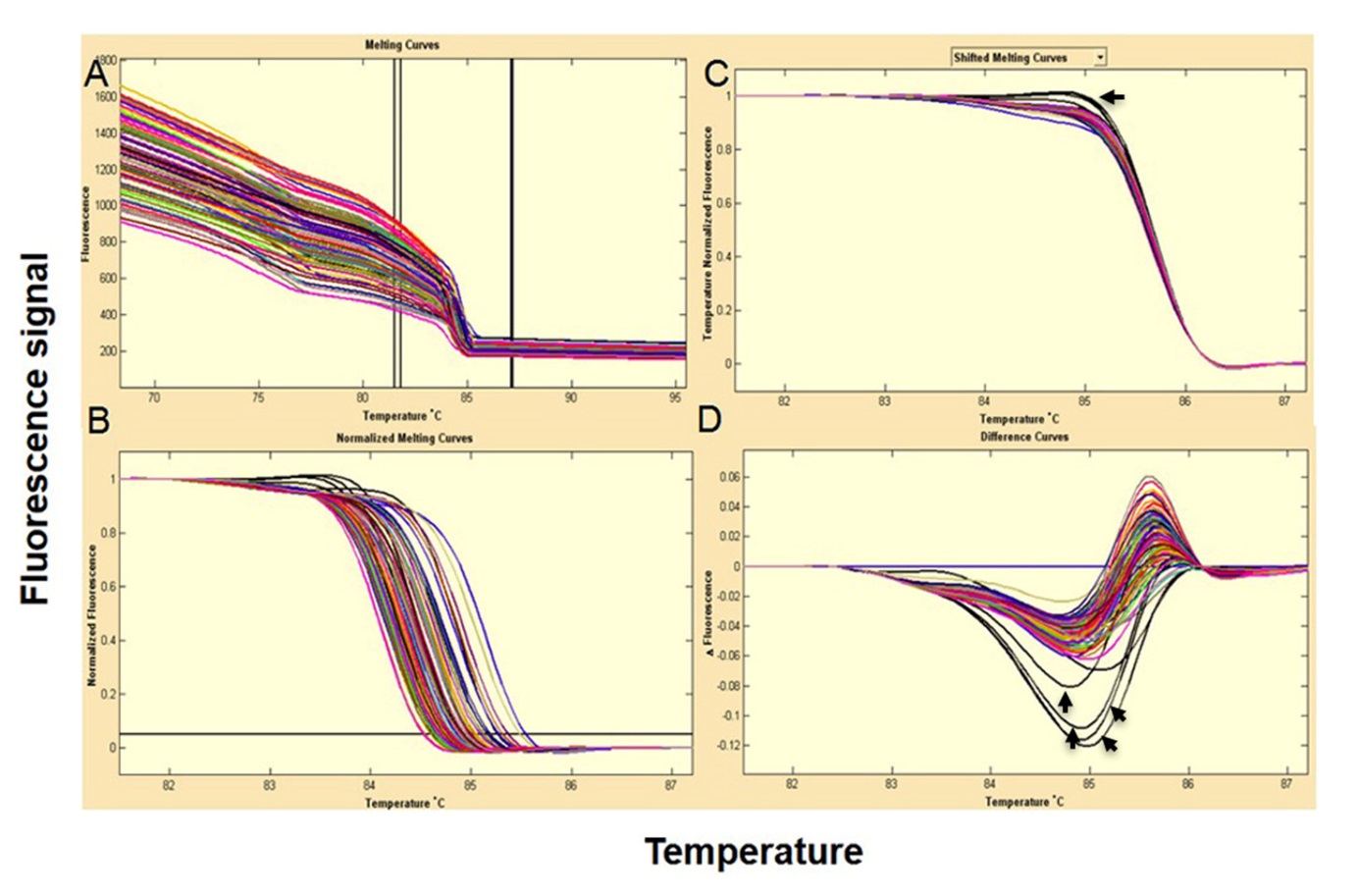
****Fig. S2. High Resolution Melting analysis of second round PCR products of 96 4x-fold pooled samples****. The figure shows the analysis of the amplicons containing known point mutation (G-A) in flc-1. (A). the original total fluorescence for 96-samples captured by the light scanner. (B). normalizing curves by changing the black bars in figure A. (C). Normalized temperature-shifted curves, the black curves pointed by arrow containing flc1 mutants (G->A) in corresponding wells and (D) difference curves with G->A point mutations highlighted in black.*

***Table S1*.** ***Primer sequences used to optimize PCR and HRM****.*

| Primer name | Gene |  | Primer sequences |
| --- | --- | --- | --- |
| Y1SP1F | *NcFLC* | Forward | AACATGCCGATGATCTTAAAGC |
| Y1SP1R |  | Reverse | GAACGAGGGAATCGACACTTAC |
| bzip19P1_F | *NcbZIP19* | Forward | AAATCGTTCTCTACGTCCATGTCT |
| bzip19P1_R |  | Reverse | TCAAAATCGACAATTTCTAAGAAGC |
| bzip19BP2_F |  | Forward | GAGTACGGTTACTTTGTTGGTGGT |
| bzip19BP2_R3 |  | Reverse | ATACTTTCGAACCGCTTCTCGATTC |
